# The Kunitz-Like Modulatory Protein Haemangin Is Vital for Hard Tick Blood-Feeding Success

**DOI:** 10.1371/journal.ppat.1000497

**Published:** 2009-07-10

**Authors:** M. Khyrul Islam, Naotoshi Tsuji, Takeharu Miyoshi, M. Abdul Alim, Xiaohong Huang, Takeshi Hatta, Kozo Fujisaki

**Affiliations:** 1 Laboratory of Parasitic Diseases, National Institute of Animal Health, National Agricultural and Food Research Organization, Tsukuba, Ibaraki, Japan; 2 Department of Emerging Infectious Diseases, School of Veterinary Medicine, University of Kagoshima, Kagoshima, Japan; Stanford University, United States of America

## Abstract

Ticks are serious haematophagus arthropod pests and are only second to mosquitoes as vectors of diseases of humans and animals. The salivary glands of the slower feeding hard ticks such as *Haemaphysalis longicornis* are a rich source of bioactive molecules and are critical to their biologic success, yet distinct molecules that help prolong parasitism on robust mammalian hosts and achieve blood-meals remain unidentified. Here, we report on the molecular and biochemical features and precise functions of a novel Kunitz inhibitor from *H. longicornis* salivary glands, termed Haemangin, in the modulation of angiogenesis and in persistent blood-feeding. Haemangin was shown to disrupt angiogenesis and wound healing via inhibition of vascular endothelial cell proliferation and induction of apoptosis. Further, this compound potently inactivated trypsin, chymotrypsin, and plasmin, indicating its antiproteolytic potential on angiogenic cascades. Analysis of Haemangin-specific gene expression kinetics at different blood-feeding stages of adult ticks revealed a dramatic up-regulation prior to complete feeding, which appears to be functionally linked to the acquisition of blood-meals. Notably, disruption of Haemangin-specific mRNA by a reverse genetic tool significantly diminished engorgement of adult *H. longicornis*, while the knock-down ticks failed to impair angiogenesis *in vivo*. To our knowledge, we have provided the first insights into transcriptional responses of human microvascular endothelial cells to Haemangin. DNA microarray data revealed that Haemangin altered the expression of 3,267 genes, including those of angiogenic significance, further substantiating the antiangiogenic function of Haemangin. We establish the vital roles of Haemangin in the hard tick blood-feeding process. Moreover, our results provide novel insights into the blood-feeding strategies that enable hard ticks to persistently feed and ensure full blood-meals through the modulation of angiogenesis and wound healing processes.

## Author Summary

Ticks are notorious ectoparasites that exclusively feed on a host's blood for a period of 10 days or longer. Upon blood-feeding, an adult female tick gains 100–200 times its body weight compared to its pre-feeding stage. Despite the host's armoury of rejection mechanisms, ticks manage to remain attached until a full blood-meal is ensured. The molecular machineries that make the tick a success with its feeding, however, remain unknown. We demonstrate that the Kunitz-like protein Haemangin, identified from the salivary glands of the tick *Haemaphysalis longicornis*, plays vital roles in blood-feeding success. Using both cell- and chick embryo–based bioassays, we have shown that Haemangin efficiently disrupted angiogenesis and wound healing processes, enabling ticks to remain attached and allowing persistent feeding. Additionally, in a rabbit model, we reveal that an elevated expression of Haemangin is associated with the acquisition of full blood-meals. Importantly, Haemangin-knockdown ticks fail to prevent angiogenesis in the host's tissues and consequently achieve only a poor blood-meal as compared to normal ticks. We conclude that Haemangin is vital for ticks' survival and can be a novel therapeutic target against ticks and tick-borne diseases, including tumor angiogenesis.

## Introduction

Angiogenesis or neovascularization, the formation of new blood vessels from pre-existing ones, is involved in a variety of physiological processes, such as corpus luteum formation, embryonic development, and wound healing [1],[2]. Also, angiogenesis plays a vital role in the development and progression of various pathological conditions, including rheumatoid arthritis, diabetic retinopathy, and tumor metastasis [3]. The process of angiogenesis involves activation and release of angiogenic factors, release of proteolytic enzymes to degrade extracellular matrix protein (ECM), migration and proliferation of endothelial cells, and microvessel formation [4]. Pericellular proteinases, comprised of membrane-type matrix metalloproteinases (MT-MMPs), serine proteinases (SPs), and membrane-bound aminopeptidases, are known to play key roles in angiogenesis [5].

The proteinase inhibitors of SPs belonging to the Kunitz, Kazal, α-macroglobulin, and serpin families play critical roles in physiological and pathophysiological states such as coagulation, intravascular fibrinolysis, wound healing, angiogenesis, and tumor metastasis [6],[7]. The best studied serine proteinase inhibitors (SPIs) to date in angiogenesis, tumor invasion, and metastasis are the plasminogen activator inhibitor-1 (PAI-1), PAI-2 [8], and maspin [9]. Kunitz-type SPIs such as tissue factor pathway inhibitor (TFPI) inhibit the proliferation of basic fibroblast growth factor (bFGF)-induced endothelial cells (ECs) in addition to their inhibitory activity against tissue factor–mediated blood coagulation cascade [10]. Snake venoms contain many unique proteins, including Kunitz-type SPIs [11]. These Kunitz proteins are potent inhibitors of trypsin, chymotrypsin, kallikrein, and plasmin; however, little is known about their physiologic roles in angiogenesis and angiogenesis-dependent diseases such as cancer.

Hard ticks are notorious ectoparasites that feed on blood for quite a long period (e.g., 10 days or more) in contrast to fast-feeder soft ticks, which usually feed for an hour [12]. Tick feeding is a complex process and involves severe tissue damage caused by the probing actions of barbed mouthparts and release of salivary secretions to the feeding lesions, leading to host's haemostatic, inflammatory, and immune responses. However, despite the host's armoury of rejection mechanisms, the ticks manage to remain attached and achieve engorgement [12],[13]. Increasing evidence suggests that success in blood-feeding relies on a pharmacy of chemicals located in the salivary glands (SGs) [14]. A recent study has reported that the saliva of the hard tick *Ixodes scapularis* is a negative modulator of angiogenesis and wound healing [15], though the specific molecular component(s) is yet to be identified. Moreover, the implications of hard-tick-modulated angiogenesis and wound healing in persistent blood-feeding remain unknown. To this end, we have looked for a novel salivary-specific molecule(s) of interest in the hard tick *Haemaphysalis longicornis*. We have selected a full-length cDNA that shows moderate sequence homologies with Kunitz-type proteinase inhibitors from the SG-specific expressed sequence tag (EST) library of adult female *H. longicornis* [16] for its functional analysis. Here, we show that an *Escherichia coli*–expressed recombinant protein, herein called Haemangin, inhibits trypsin, chymotrypsin, and plasmin. Also, we provide novel evidence that this Haemangin-like modulatory protein is the key to the modulation of angiogenesis and angiogenesis-dependent wound healing during persistent blood-feeding and is vital for hard ticks in achieving host blood-meals.

## Results

### Molecular Characterization of Haemangin

**Figure 1:**
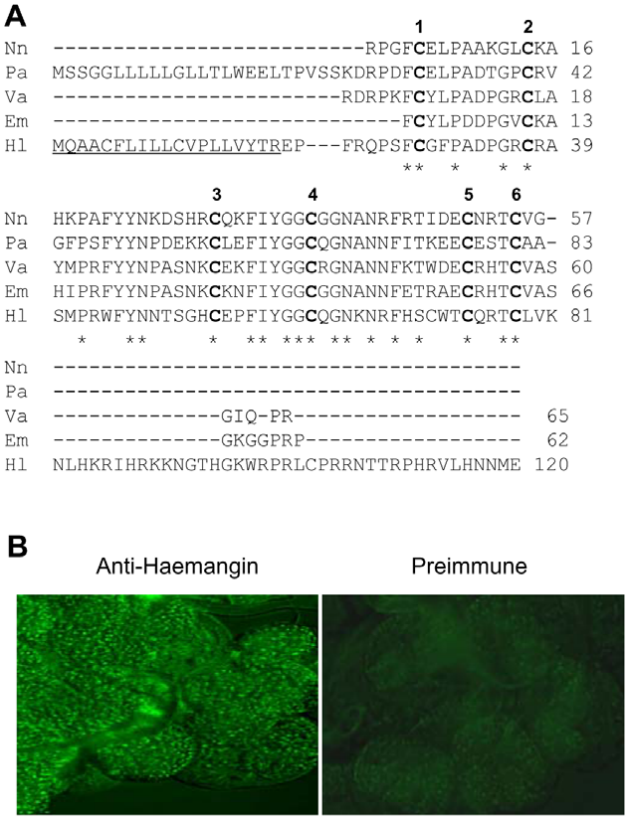
Primary structure and expression of Haemangin. (A) Sequence alignment of Haemangin with other venom basic proteinase inhibitors belonging to the BPTI/Kunitz family of SPIs. CLUSTALW (1.83) alignment of BPTI/Kunitz-type SPIs: Nn, *N. naja* (P20229); Pa, *Pseudodechis australis* (AAT45402); Va, *V. ammodytes ammodytes* (P00992); Em, *Eristocophis macmahonii* (P24541); Hl, *H. longicornis* (AB434485). The signal peptides are underlined. Identical amino acid residues are marked with asterisks. The six conserved cysteine residues are in bold with numbers. Dashes indicate gaps inserted to optimize the alignment. (B) Endogenous expression of Haemangin in adult *H. longicornis* SGs by immunofluorescence. Freshly obtained SGs of a partially fed adult tick were washed in 0.1% PBS-T, fixed in acetone, and incubated with primary anti-Haemangin antibody. FITC-labeled goat anti-mouse IgG was used as a secondary antibody (left panel). *H. longicornis* SGs treated with mouse pre-immune sera are regarded as control (right panel).

The composite full-length Haemangin cDNA sequence was 583 nucleotides long with a single open reading frame (ORF) of 363 bases. The ORF coded for a protein of 120 amino acids, including a signal peptide of 19 residues (data not shown). The putative mature protein has a molecular mass of 14,157 Da and an isoelectric point (p*I*) of 10.65. The deduced protein has three potential *N*-glycosylation sites. The unique primary structure of the predicted protein consists of 10 cysteines forming 5 disulfide bonds, a lysine- and histidine-rich carboxy terminus, and a single Kunitz-like inhibitory domain that belongs to bovine pancreatic trypsin inhibitor (BPTI)/Kunitz family of SPI, as detected with Scan-Prosite program. A BLASTX analysis of the translated product deduced from the ORF revealed that Haemangin shares the greatest sequence identity (56%) with kalicludine 1, a K^+^ channel inhibitor that belongs to the BPTI/Kunitz family of SPI, obtained from the toxin of *Anemonia sulcata* (accession number AAB35413). Haemangin also shares 51% sequence identity with venom trypsin inhibitor isolated from the venom of *Naja naja* (P20229), 49% with isoinhibitor K, a trypsin-kallikrein inhibitor K from *Helix promatica* (P00994), 48% with TFPI from *Homo sapiens* (AA089075), and 47% with venom basic protease inhibitor I of *Vipera ammodytes ammodytes* (P00992). A comparison of the deduced amino acid sequence of Haemangin with those of venom basic proteinase inhibitors belonging to the BPTI/Kunitz family of SPIs is shown (Figure 1A). Alignment data revealed that out of 5 disulfide bonds, 3 were conserved. The predicted signal peptides, however, were not conserved. Phylogenetic data revealed that Haemangin within the BPTI/Kunitz family of SPIs forms a separate single cluster and is distantly related to Kunitz-type inhibitors of snake-venom origin (data not shown).

Haemangin-specific transcript profiling at different blood-feeding stages was analyzed by quantitative RT-PCR. Data revealed that the target transcript was up-regulated as blood-feeding progressed. A dramatic increase of Haemangin expression was observed at 96 h of blood-feeding prior to acquisition of a full blood-meal, which then sharply declined to a minimal level (data not shown), indicating that Haemangin plays a role in the acquisition of blood-meals.

The endogenous expression of Haemangin in SGs of a partial-fed adult *H. longicornis* was shown to be localized in the salivary acini by immunofluorescence staining (Figure 1B, left panel), but was not detected in the SGs treated with pre-immune mouse sera (Figure 1B, right panel).

### Inhibition of Vascular Sprouting in Chick Aorta

**Figure 2:**
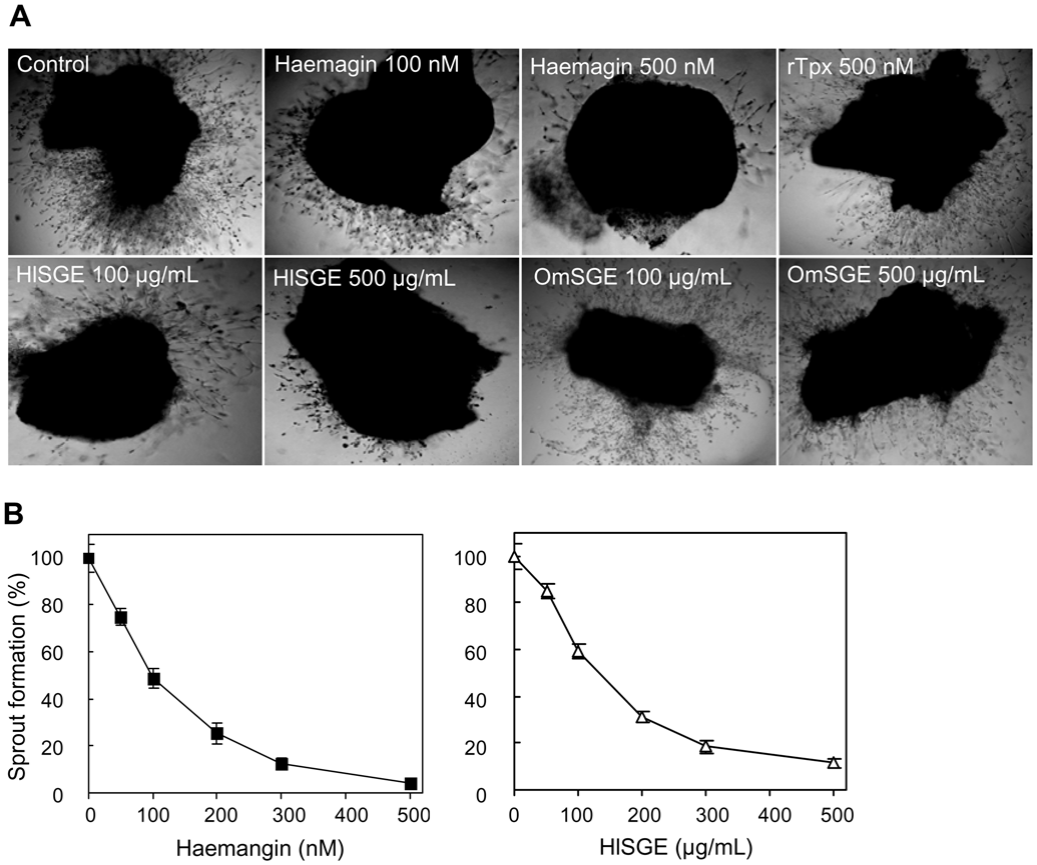
Inhibition of vascular sprout formation in chick aorta *in vitro*. (A) Chick aortic rings (∼1.0 mm) obtained from 12-day-old embryos were placed in a 96-well tissue culture plate coated with 20 µl Matrigel. Then, 100 µl of EBM-2 media supplemented with penicillin and streptomycin was added, followed by the addition of an increasing concentration of Haemangin or HlSGE/OmSGE. The plate was incubated at 37°C and at 5% CO_2_. Vessel sprout formation was observed for 3 days and images were taken (magnification×100). (B) Dose-dependent response curves. Data on vessel sprout formation in the presence of an increasing concentration of Haemangin or HlSGE compared to media alone (control, 100%) are expressed as percentages. Error bars indicate the standard error of the mean (n = 5).

A chick aortic ring assay was employed to examine whether Haemangin inhibited angiogenesis *in vitro*. The absence of Haemangin (control) allowed the formation of dense vascular sprouts in aortic ring explants, while its presence (∼500 nM) potently inhibited vascular sprouting in a dose-dependent manner (Figure 2A). HlSGE (∼500 µg/ml) also strongly inhibited vascular sprouting in a dose-response manner. The IC_50_ for Haemangin and HlSGE was 100.81 nM and 129.63 µg/ml, respectively. A dose-dependent inhibition curve of vascular sprouting is shown (Figure 2B). We also observed that transfected *E. coli* lysate (∼100 µl/well) but not non-transfected *E. coli* lysate (∼100 µl/well) exerted an inhibitory activity (data not shown). Addition of rTpx (500 nM) did not show any inhibitory effect on vascular sprouting (Figure 2A). OmSGE (∼500 µg/ml) completely failed to inhibit vascular sprouting (Figure 2A).

### Inhibition of Capillary Tube Formation

**Figure 3:**
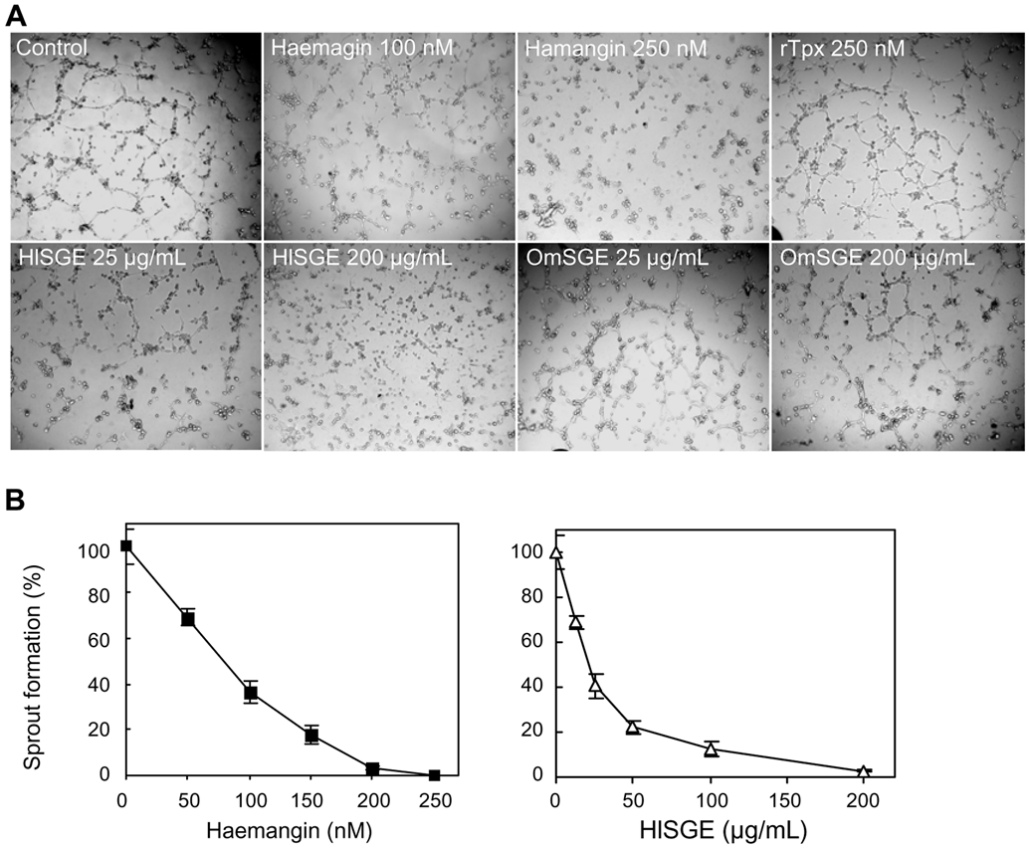
Inhibition of capillary tube formation *in vitro*. (A) HUVECs were seeded (7.5×10^3^ cells/well) into a 96-well tissue culture plate coated with 50 µl Matrigel. Then, an increasing concentration of Haemangin or HlSGE/OmSGE was added. Cells were incubated in HUVEC growth medium at 37°C, 5% CO_2_. Tube formation was observed for 6 h and images were taken (magnification×100). (B) Dose-dependent response curves. Data on tube formation in the presence of an increasing concentration of Haemangin or HlSGE compared to media alone (control, 100%) are expressed as percentages. Error bars indicate the standard error of the mean (n = 3).

To further evaluate the potential of Haemangin in inhibiting angiogenesis, we used human umbilical vein endothelial cells (HUVECs) in a 96-well plate to examine Matrigel-induced morphogenic differentiation of ECs into capillary-like tubes. Data revealed that in the absence of Haemangin, HUVECs were rapidly organized, forming a number of well-defined capillary-like structures in Matrigel (Figure 3A). The presence of Haemangin (∼250 nM) strikingly inhibited tube formation. HlSGE (∼200 µg/ml) also inhibited tube formation in a dose-dependent manner. Tube formation was also inhibited by transfected *E. coli* lysate (∼100 µl/well), but not by non-transfected *E. coli* lysate (∼100 µl/well) (data not shown) or by rTpx (250 nM). Of interest, OmSGE (∼200 µg/ml) did not inhibit tube formation. Dose-dependent inhibition curves of tube formation are shown in Figure 3B. The IC_50_ for Haemangin and HlSGE was 55.82 nM and 20.13 µg/ml, respectively.

### Inhibition of Angiogenesis in CAM

**Figure 4:**
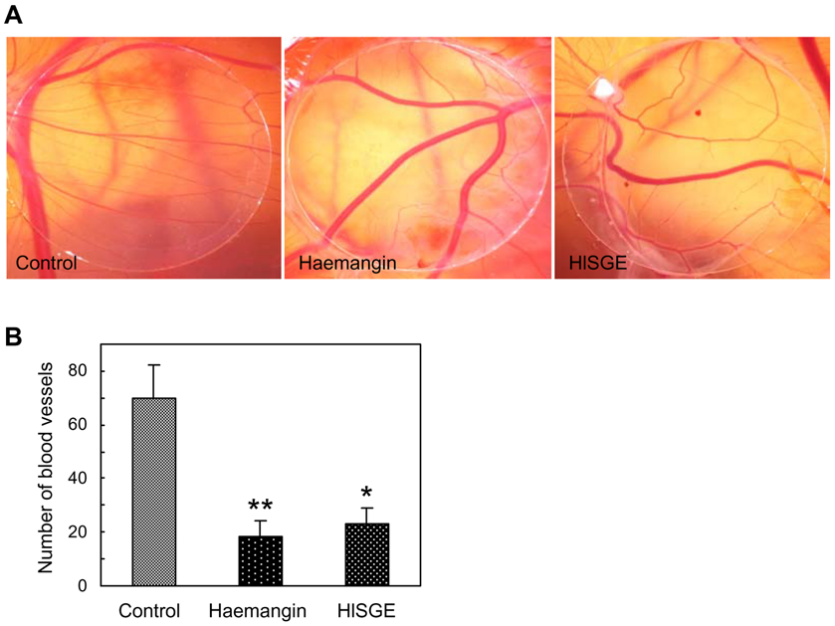
Inhibition of angiogenesis in CAM *in vivo*. (A) Thermanox discs, 13 mm in diameter, loaded either with PBS only or test compounds (Haemangin 500 ng/embryo and HlSGE 2.5 µg/embryo) were applied to CAMs of 8-day-old chick embryos. After 48 h of incubation at 37°C, negative or positive responses were assessed. Images were taken using a digital camera (Canon). (B) Quantification of inhibition of angiogenesis by Haemangin and HlSGE. Numbers of vessels were from 7–12 CAMs in each group. **p<0.001 and *p<0.01 versus control. Error bars indicate the standard error of the mean.

Haemangin distinctly abolished angiogenesis from the preexisting blood vessels, while chick chorioalantoic membranes (CAMs) treated with PBS alone revealed normal angiogenesis (Figure 4A). HlSGE also inhibited angiogenesis in CAMs. Data on quantitative analysis showed that Haemangin at 500 ng/disc and HlSGE at 2.5 µg/disc inhibited approximately 80% and 72% neovascularization, respectively (Figure 4B).

### Inhibition of Cell Proliferation and Wound Healing

**Figure 5:**
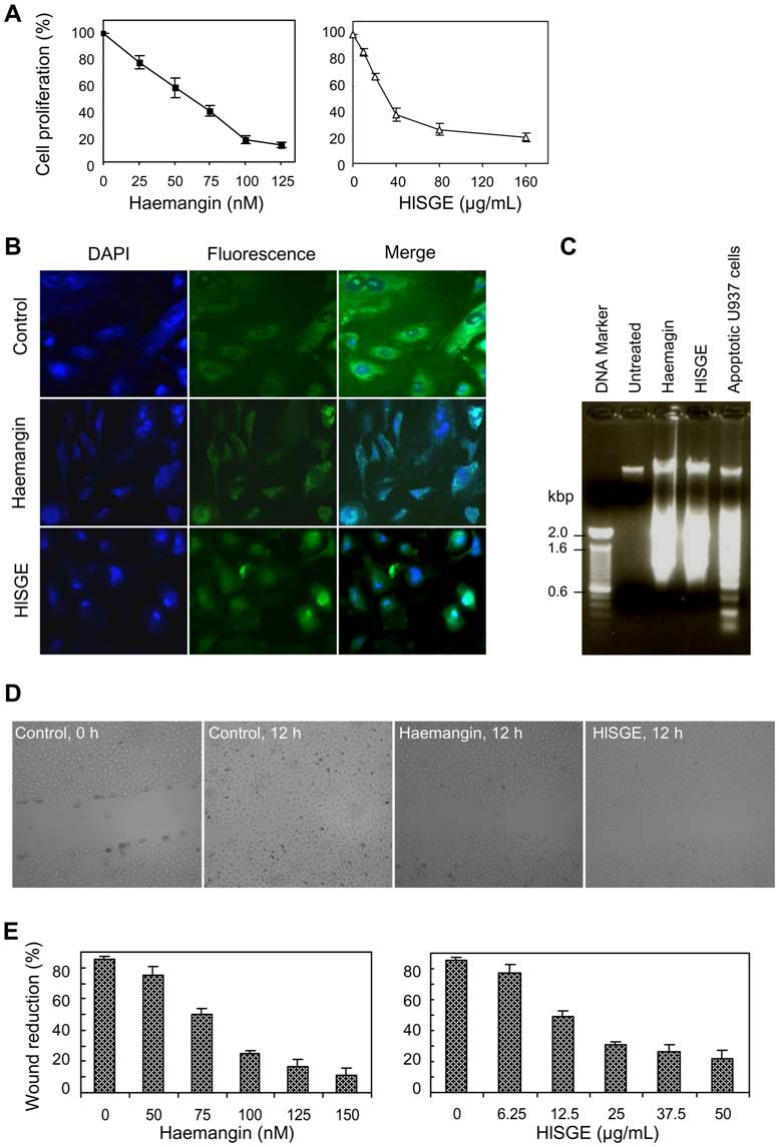
Inhibition of cell proliferation and wound healing. (A) Cells were seeded (3×10^3^ cells/well) into a 96-well tissue culture plate and were incubated in HUVEC growth medium in the absence or presence of Haemangin or HlSGE at 37°C, 5% CO_2_ for 72 h. The numbers of cells in each well were determined after the addition of MTS solution, and inhibition of cell proliferation (% of control) was then estimated. Error bars indicate the standard error of the mean (n = 3). (B) Induction of apoptosis of ECs. HUVECs were seeded (1×10^5^ cells/well) into a 6-well tissue culture plate and were incubated in the absence or presence of Haemangin (250 nM) or HlSGE (25 µg/ml) for 48 h as in (A). Cells were stained with Hoechst 33258 (magnification×200). (C) DNA ladder formation in HUVECs. Cells were seeded (2.5×10^5^ cells/flask) into a T-25 tissue culture flask and were incubated in the absence or presence of Haemangin (250 nM) or HlSGE (25 µg/ml) for 48 h as in (A). Cytoplasmic DNA was isolated and analyzed using 1% agarose gel electrophoresis. (D) Inhibition of wound healing *in vitro*. HUVECs were seeded (1.25×10^5^ cells /well) into a 6-well tissue culture plate and were grown to confluency in HUVEC growth medium. An increasing concentration of either Haemangin or HlSGE was added at the time of scratching, and the wound healing process was observed for 12 h. Wounds were treated with Haemangin (150 nM) and HlSGE (50 µg/ml), respectively (magnification×50). (E) Quantification of wound reduction by Haemangin and HlSGE. Error bars indicate the standard error of the mean (n = 3).

Haemangin potently blocked proliferation of HUVECs in a dose-dependent manner with an IC_50_ of 20.07 nM (Figure 5A). Consistent results were obtained with HlSGE with an IC_50_ of 10.11 µg/ml (Figure 5A). The cell cytotoxicity effect of both Haemangin and HlSGE was determined by measuring the lactate dehydrogenase (LDH) activity. Neither Haemangin nor HlSGE showed any cytotoxic effect at concentrations used for cell proliferation assays (data not shown), suggesting that Haemangin is non-toxic to mammalian cells.

To investigate whether Haemangin-induced inhibition of cell proliferation is associated with apoptosis, HUVECs were incubated with Haemangin and were stained with Hoechst 33258 for observing changes characteristic of apoptosis. Haemangin markedly induced apoptosis of HUVECs at a concentration of 250 nM (Figure 5B). The nuclei of living cells stained with Hoechst 33258 are shown as intact, with smooth and regular contour, while the apoptotic cells show shrinkage and rounding, with highly condensed, marginated, and fragmented nuclei, which are the hallmarks of apoptosis [17]. HlSGE also caused apoptosis at a concentration of 25 µg/ml (Figure 5B). A DNA-ladder assay was performed to detect the typical DNA ladder, which is the characteristic of apoptotic cells [17]. DNA extracted from Haemangin- and HlSGE-treated cells showed an apparent laddering pattern of DNA fragmentation (Figure 5C).

We have hypothesized that hard ticks evolved novel strategies to suppress wound healing to avoid rejection by the host. To test this hypothesis, we examined the ability of Haemangin to thwart wound healing using artificially created wounds in a monolayer of HUVECs (Figure 5D). In the absence of Haemangin, wound healing was completed within 12 h (Figure 5D). Notably, Haemangin dose-dependently inhibited wound healing with an IC_50_ of 49.49 nM (Figure 5E). Similar results were obtained with HlSGE with an IC_50_ of 10.49 µg/ml (Figure 5E).

### Inhibition of Serine Proteinases

**Figure 6:**
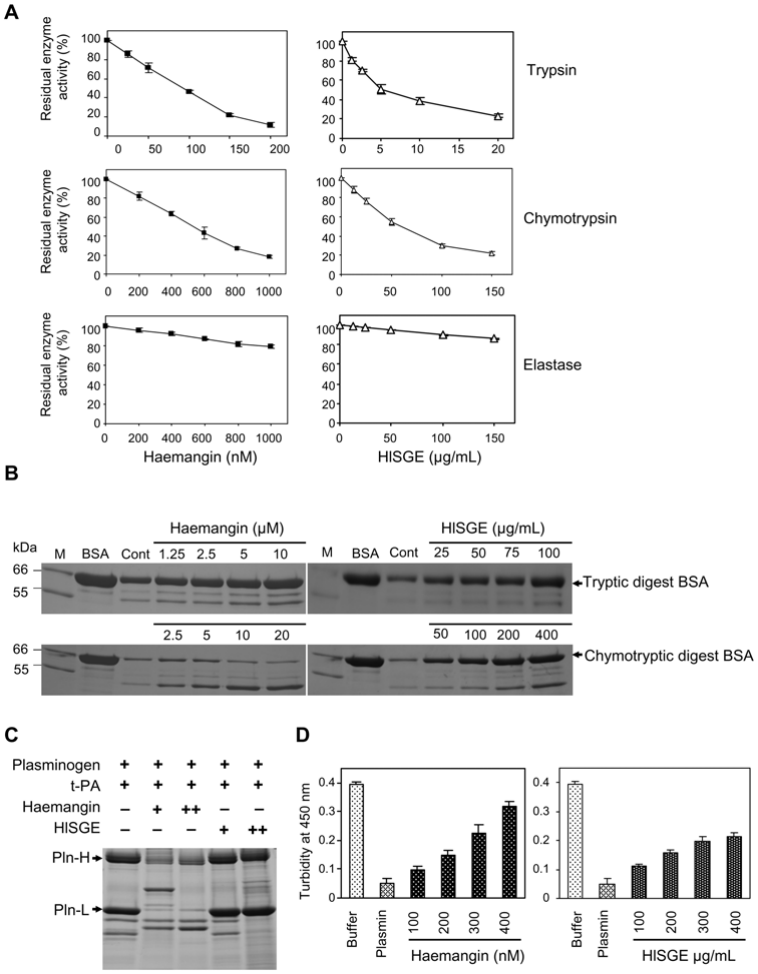
Inhibition of serine proteinases. (A) Inhibition of hydrolytic activity of trypsin, chymotrypsin, and elastase on synthetic substrates. Enzyme inhibition assays were performed using fluorogenic substrates by measuring the residual hydrolytic activity after pre-incubation with increasing concentrations of Haemangin or HlSGE. The final concentrations of enzyme/substrate used were as follows: trypsin (5.6 nM)/Substrate (10 µM), chymotrypsin (5 µM)/Substrate (10 µM), and elastase (51 nM)/Substrate (10 µM). Each enzyme was pre-incubated with an increasing concentration of Haemangin at 37°C for 30 min. After pre-incubation of enzyme and inhibitor, appropriate fluorogenic substrate was added. The total volume of reaction mixture was 200 µl in individual wells in 96-well flat bottomed plates. The residual enzyme activity was measured by reading the fluorescence over time at excitation and emission wavelengths of 360 and 460 nm, respectively. PMSF was used as a positive control. Percent inhibition by Haemangin is assessed by the following formula: % inhibition = (1–inhibited rate/uninhibited rate)×100. Error bars indicate the standard error of the mean (n = 3). (B) Inhibition of BSA proteolysis by trypsin and chymotrypsin. Proteolysis inhibition assays were done by pre-incubating the enzymes with increasing concentration of Haemangin or HlSGE in 20 mM Tris-HCl (pH 8.0) in a total reaction mixture of 40 µl for 1 h at 37°C. Then, BSA (500 µg/ml) was added to the inhibited and uninhibited enzymes (250 µg/ml) (control) and was further incubated overnight at 37°C. The following morning, tryptic digests were electrophoresed in 12.5% SDS-PAGE under reducing condition. Molecular weight markers in kDa (lane M) are shown on the left. (C) Effects of Haemangin on plasmin degradation during fibrinolysis. Fibrinogen (8 µM) and thrombin (0.83 NIH unit/ml) were incubated in a buffer containing 50 mM Tris-HCl (pH 7.5), 100 mM NaCl, and 5 mM CaCl_2_ at 25°C for 30 min. After the fibrin was completely polymerized, lysis of fibrin polymer was initiated by the addition of 20 µM plasminogen and 15 nM t-PA. Reactions were started in the absence or presence of 200 nM (+) and 400 nM (++) Haemangin, and 150 µg/ml (+) and 300 µg/ml (++) HlSGE separately. The reaction mixtures were incubated overnight at 25°C and were subjected to 8% SDS-PAGE under reducing condition. (D) Inhibition of plasmin-dependent fibrin polymer lysis. Fibrinogen (8 µM) and thrombin (0.83 NIH unit/ml) were incubated in a buffer mentioned in (C). After fibrin polymerization was completed, a 100 µl buffer alone or buffer containing plasmin (1.5 µM) without or with an increasing concentration of Haemangin or HlSGE was added to initiate fibrinolysis. Error bars indicate the standard error of the mean (n = 3).

Haemangin dose-dependently inhibited trypsin and chymotrypsin, showing maximum inhibition of 90% and 82% with an IC_50_ of 64.65 nM and 294.61 nM, respectively (Figure 6A). Haemangin poorly inhibited elastase (21%) (Figure 6A). HlSGE also potently inhibited the activity of trypsin and chymotrypsin and slightly inhibited elastase by 77%, 78%, and 14%, respectively (Figure 6A). The IC_50_ was 6.43 µg/ml and 51.71 µg/ml for trypsin and chymotrypsin, respectively. Data on BSA proteolysis inhibition assays showed that Haemangin inhibited BSA proteolysis by trypsin and chymotrypsin in a dose-dependent fashion (Figure 6B). Consistent results were obtained with HlSGE (Figure 6B).

Haemangin was able to efficiently stimulate degradation of both the plasmin heavy and light chains during fibrinolysis (Figure 6C). However, relatively little activity was detectable with HlSGE (Figure 6C). To further evaluate whether Haemangin directly inhibit plasmin activity, *in vitro* fibrin polymer lysis assays were conducted. A fibrin polymer was initially prepared and then fibrinolysis was initiated by the addition of plasmin in the absence or presence of increasing concentrations of Haemangin. An increase in turbidity was detected as polymerization of fibrin proceeded (data not shown), and reached a maximum value when a stable fibrin polymer was formed (Figure 6D). A plasmin-induced fibrinolysis yielded a clear suspension of the fibrin polymer and exhibited a decrease in turbidity (Figure 6D). Haemangin strongly inhibited plasmin-dependent fibrinolysis while HlSGE moderately blocked fibrinolytic activity of the added plasmin (Figure 6D), suggesting that Haemangin is able to inhibit the proteolytic activity of plasmin on cross-linked fibrin polymer.

### Modulation of Gene Expression Profiles

An attempt was made to examine the global gene expression profiles of HUVECs stimulated with Haemangin to gain a precise understanding of the molecular signaling cascades that facilitate feeding and engorgement of hard ticks. By comparing the expression profiles between Haemangin-exposed and nonexposed HUVECs, gene ontology data based on molecular functions revealed a total of 506 responsive genes out of 35,000 genes expressed on the microarray chips. Of 506 altered genes, 317 were up-regulated and 189 were down-regulated genes (Figures S1 and S2). On the other hand, gene ontology data based on biological processes demonstrated a total of 2,761 induced genes, of which 2,199 genes were up-regulated and 562 were down-regulated (Tables S1 and S2).

### Modulation of Tick Blood-Feeding

**Figure 7:**
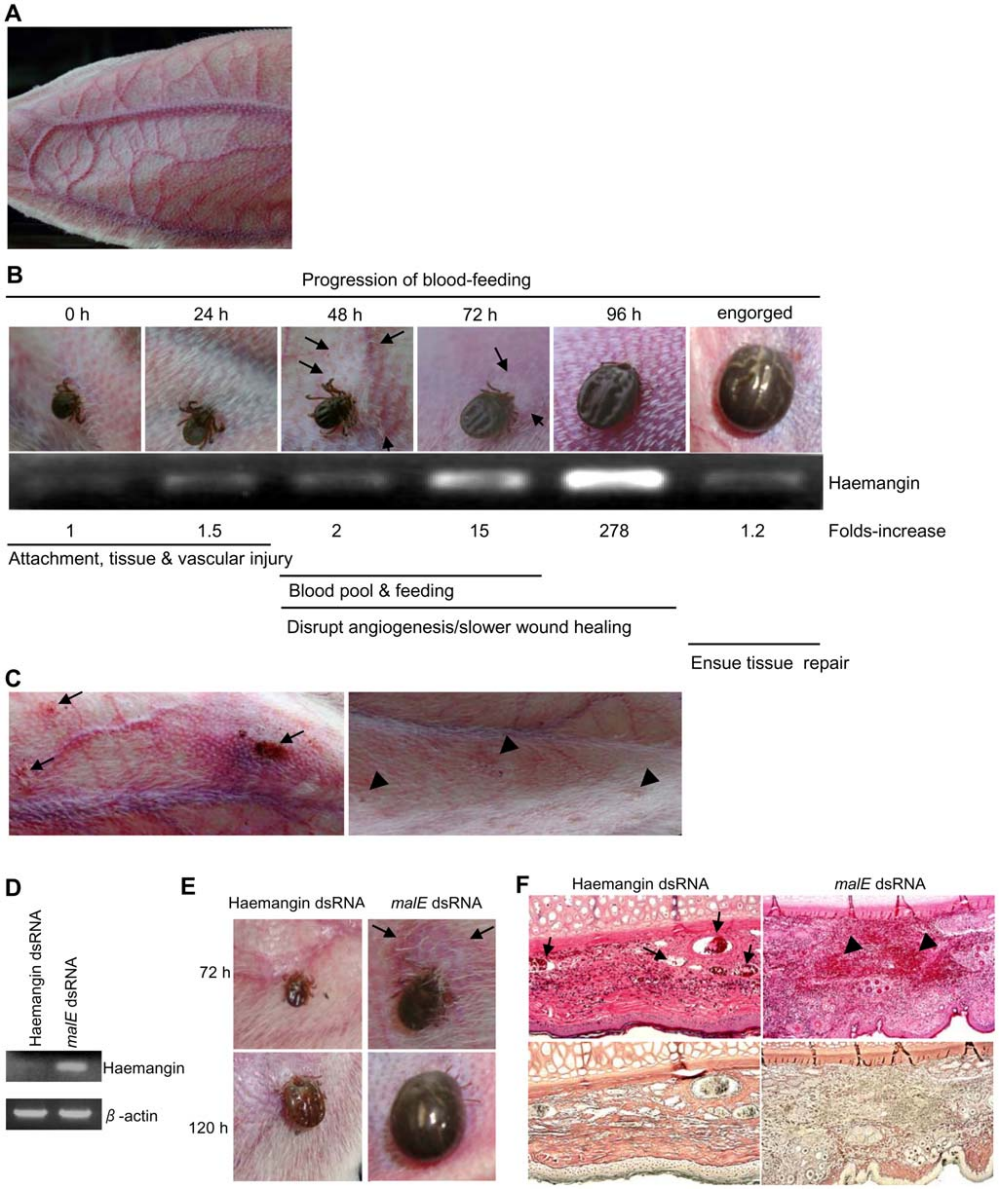
Haemangin's function in blood-feeding *in vivo*. (A) A naïve rabbit's ear as source of blood-meals. (B) Blood-feeding progression and engorgement of adult *H. longicornis*. Semi-quantitative RT-PCR was performed. Expression of Haemangin-specific mRNA is shown. Arrows indicate areas of blood pool. (C) A rabbit ear showing feeding wounds after the engorged ticks dropped off the ear (arrows; left panel) and repaired wounds on day 10 post-dropping (arrowheads; right panel). (D) Gene silencing of Haemangin by RNA interference. Ticks were injected either with *E. coli malE* dsRNA (control) or with Haemangin dsRNA. RT-PCR analysis was performed. Expression of Haemangin-specific mRNA is shown. β-actin is shown as an internal control. (E) Effect of targeted gene silencing on blood-feeding by adult ticks. Images were captured at 72 h and 120 h post-feeding. Arrows indicate areas of blood pool. (F) Effect of targeted gene silencing on angiogenesis *in vivo*. H&E- and silver nitrate–stained sections of a rabbit ear at 120 h tick post-feeding, revealing capillaries and venules (arrows) and area of blood pool (arrowheads) (magnification×100).

To explore Haemangin's function *in vivo*, adult *H. longicornis* were allowed to feed on rabbits (Figure 7A). Tick feeding progression and engorgement were monitored and transcriptional responses at different feeding stages were analyzed by semi-quantitative RT-PCR. Data were consistent with that of quantitative RT-PCR (Figure 7B). Tick feeding progression is summarized in Figure 7B. Haemangin expression was steadily up-regulated with the progression of feeding, showing a dramatic peak prior to acquisition of full blood-meals, and then sharply declined upon engorgement. Healing of the feeding wounds commenced immediately after ticks dropped off the host (Figure 7C, left panel), and completed within 10–14 days (Figure 7C, right panel).

To validate gene functions *in vivo*, an RNA interference (RNAi) tool was employed using Haemangin-specific double-stranded RNA (dsRNA). Data revealed very scanty expression of Haemangin-specific mRNA upon feeding, indicating that gene knockdown was successful (Figure 7D). Notably, knockdown ticks completely failed to make blood pools by 72 h and achieved significantly diminished engorgement by 144 h, while control ticks become engorged by 120 h, as shown by engorged body weights of 32.89±18.36 and 314.18±63.17 mg (*p*<0.001), respectively (Figure 7E). Histologically, knockdown tick-fed lesions showed an increased neovascularization compared with controls, suggesting a novel antiangiogenic role for Haemangin (Figure 7F).

## Discussion

Prior studies suggest that slower feeding hard ticks heavily rely on salivary molecules for acquisition of blood-meals [12],[13],[18]. Here, we report on a salivary Kunitz inhibitor of *H. longicornis* that modulates angiogenesis and wound healing, enabling ticks to feed and engorge. Furthermore, our results substantiate a diversified role of Kunitz proteins in angiogenesis-dependent human diseases.

We have shown that Haemangin pronouncedly disrupted angiogenesis through inhibition of EC proliferation and induction of apoptosis. These observations conform to the report of Francischetti *et al.* [15], who described similar roles for tick saliva, suggesting that tick saliva has MP activity that may cause inhibition of cell proliferation and induction of apoptosis, although the salivary molecule(s) responsible for the inhibition of angiogenesis was not confirmed. Angiogenesis is dependent on fibrinolytic MPs and plasmin, indicating that cleavage of fibrin is required for angiogenic cascades [19],[20]. Angiogenesis is characterized by EC proliferation, migration, and capillary tube formation, and is dependent on cell adhesion molecules and proteinases [19],[21]. In contrast, inhibition of cell proliferation and induction of apoptosis are among the most effective means of disruption of angiogenesis. Proteolytic degradation of ECM and the vascular basement membrane, and remodeling of the ECM components is essentially required to allow ECs to migrate into and invade the surrounding stroma [5],[22],[23]. However, excessive degradation of ECM is incompatible with efficient migration of EC [24]. It has been anticipated that the maintenance of a certain degree of ECM integrity is indeed essential for capillary morphogenesis [25]. We hypothesized that Haemangin exerts an antiangiogenic function through the inhibition of proteolytic cascades, although we cannot rule out alternative roles, such as the inhibition of cell adhesion mechanisms by inactivating angiogenic growth factors. Plasmin cleaves fibrinogen and fibrin, and activates various MMPs. Once activated, plasmin can act by itself on ECM proteins and activates MMP-1, MMP-2, MMP-3, and MMP-9 [26],[27]. Furthermore, in addition to MT1-MMP, plasmin has been indicated to be involved in endothelial tube formation in a fibrin matrix [19],[20],[28]. Of note, the ECM is known to act as a sequestration for angiogenic growth factors, for example, vascular endothelial growth factor (VEGF), bFGF, and transforming growth factor (TGF)-β1, which can be released upon proteolytic degradation of the ECM [29], suggesting that plasmin is critical for angiogenesis. Overall, our data indicate that Haemangin-induced plasmin proteolysis plays an important role in the inhibition of angiogenesis during blood-feeding by hard ticks. For the suppression of tumor angiogenesis, however, a tightly controlled proteolysis by proteinase inhibitors (for example, uPA, plasmin, and probably MPs) is required [25].

Growth factors and their receptors are important mediators of angiogenesis. The VEGF and bFGF are the two most potent angiogenic factors that stimulate angiogenic cascades independently [30]. Growth factors specifically bind and interact with their receptors, which are all tyrosine kinases, present on the EC surface, and in turn activate different signal transduction pathways, leading to alteration of the transcription of genes involved in cell migration, apoptosis, proliferation, and tube formation [31]. Haemangin markedly induced apoptosis and blocked EC proliferation directly, indicating that it may simultaneously alter expression of angiogenic factors and/or inactivate cell surface receptors. To explore molecular pathways of Haemangin-modulated angiogenesis, high-throughput studies were performed. Data revealed that Haemangin induces a wide spectrum of genes associated with various biological processes, including apoptosis, angiogenesis, and wound healing. An enhanced expression of genes in response to cytokine and chemokine activity, VEGF receptor activity, IL-6 receptor binding, and receptor tyrosine kinase binding might reflect a process of activation of inflammation and wounding, and higher demand for cell proliferation and differentiation to supply oxygen and nutrients for tissues in repairing process [32],[33]. Haemangin was also shown to repress a number of genes of angiogenic significance. Extracellular signal–regulated kinase/mitogen-activated protein kinase (ERK/MAPK) signaling plays crucial roles in diverse cellular functions, including cell proliferation, differentiation, migration, and survival [34]. Also, FGF receptor signaling is known to modulate a wide variety of cellular functions, including epithelial cell morphogenesis [35]. Adenosine 3′,5′-cyclic monophosphate (cAMP)-binding proteins directly activate Rap1, a member of the Ras superfamily of small G proteins that, in turn, stimulate and inhibit the activity of MAPK, such as B-Raf and Raf-1, respectively, leading to altered activity of ERK/MAPK that is critical for growth factor–induced cell proliferation and angiogenesis [36],[37]. Thus, Haemangin appears to utilize multiple intracellular signaling pathways to negatively regulate angiogenesis and angiogenesis-dependent wound healing.

Angiogenesis plays a central role in the wound healing process [38]. Proteolytic degradation of ECM by serine proteinases, particularly plasmin, and MMPs is crucial in this process [39]. EC proliferation is one of the important steps in angiogenesis and is critical for granulation tissue formation, a hallmark of wound healing [40]. Data presented above strongly suggest that Haemangin is capable of inhibiting different phases of wound healing, from proteolytic degradation of ECM to EC proliferation and differentiation, and eventually affects the tissue repair process. This may likely slow tick rejection and favor feeding and engorgement.

Salivary anticoagulants (Salp14 and its paralogs) and cysteine protienase inhibitors in *I. scapularis* are reported to contribute to blood-feeding success [41],[42], although their roles in angiogenesis and wound healing are still unclear. Of note, a dramatic upregulation of Haemangin-specific mRNA prior to the acquisition of full blood-meals indicated its crucial role in blood-meal acquisition. This finding, together with RNAi data, strongly supports such a role for Haemangin. Interestingly, the fast-feeder counterpart *Ornithodoros moubata* lacks both Haemangin-like Kunitz inhibitor and antiangiogenic functions, and is similar to other fast-feeder haematophagus arthropods, for example, mosquitoes and sand flies, whose SGs do not affect ECs [15]. This may likely explain the functional significance of Haemangin in blood-feeding strategies and success by slower feeding arthropods like hard ticks.

In conclusion, these results show that unlike fast-feeder arthropods, slow-feeder hard ticks evolve novel strategies to remain attached on large mammalian counterparts and achieve engorgement through the modulation of angiogenesis and wound healing processes. Furthermore, the Haemangin-like modulatory peptide(s) is considered vital for the hard tick's survival and can be a potential therapeutic target against ticks and tick-borne pathogens, including tumor angiogenesis.

## Materials and Methods

### Ticks and Tick Salivary Glands

*H. longicornis* and *O. moubata* (soft tick) were maintained in the Laboratory of Parasitic Diseases, National Institute of Animal Health (NIAH), Japan. SGs were obtained from adult *H. longicornis* at different blood-feeding stages. SGs from adult soft ticks were collected after partial blood-feeding on rabbits. Animal experimentation was done in accordance with the protocol approved by the NIAH Animal Care and Use Committee.

### Cloning and Sequencing of cDNA

Recently, our group generated a total of 8,000 ESTs from the SGs cDNA libraries of adult *H. longicornis* [16]. Of these, a full-length cDNA clone encoding a protein called Haemangin showing moderate homologies with Kunitz-type inhibitors was selected for this study. The BLAST program for alignment was used to compare the Haemangin sequence with previously reported sequences available in GenBank.

### Recombinant Proteins

The coding region of the Haemangin gene including the signal sequence was amplified by PCR. A sense primer (5′- GGAATTCCTCACTCCATGTTGTTATGTAA-3′) containing an *EcoR*I site upstream of the start codon and an antisense primer (5′-CCGAGCTCGAGACGAGAGCCATTTCGCCAACCA-3′) containing a *Xho*I site just downstream of amino acid residue were used (Promega). The purified PCR product was inserted into the *EcoR*I and *Xho*I sites of plasmid expression vector pTrcHisB (Invitrogen). The resultant plasmid was transformed into *E. coli* strain TOP10F′ (Invitrogen) cells and expressed as a fusion protein. Recombinant proteins were produced and solubilized under denaturing conditions using 20 mM sodium phosphate, 500 mM sodium chloride, and 8 M urea (pH 7.8), and then purified by histidine binding affinity chromatography (Bio-Rad) followed by dialysis against 20 mM Tris-HCl (pH 8.0) and 150 mM NaCl. The purity of Haemangin was judged by SDS-PAGE. Protein concentrations were measured with the Micro BCA Protein Assay Reagent (Pierce).

### Immunofluorescence

SGs were washed in PBS containing 0.1% Tween 20 (PBS-T) and were fixed in acetone. After fixation, they were blocked with 10% goat serum (Wako) for 1 h and were incubated with anti-mouse IgG to Haemangin overnight. FITC-conjugated goat anti-mouse IgG (Sigma) was used as a secondary antibody and the fluorescence was visualized under a Leica microscope.

### Proteinase Assay

Proteinase inhibition assays were performed by measuring the residual hydrolytic activity after preincubation with Haemangin. To detect the inhibitory activity of endogenous Haemangin, soluble extracts of *H. longicornis* SG extract (HlSGE) were prepared in 20 mM Tris-HCl (pH 8.0). The enzymes were preincubated with Haemangin or HlSGE for 30 min at 37°C. Then, appropriate substrates in a 200 µl reaction mixture consisting of 100 mM Tris-HCl (pH 8.0), 100 mM NaCl, and 20 mM CaCl_2_ in a 96-well plate were added, followed by incubation for 1 h at 37°C. Fluorogenic substrates for trypsin (Boc-Gln-Ala-Arg-MCA) (1 mM), chymotrypsin (Suc-Ala-Ala-Pro-Phe-MCA) (1 mM), and elastase (Suc-Ala-Ala-Ala-MCA) (1 mM) (Peptide Institute Inc.) were used. Substrate hydrolysis was monitored by measuring excitation and emission wavelength at 360 nm and 460 nm, respectively, over time. Percentage of inhibition by Haemangin of enzyme activity was assessed by the following formula: % inhibition = (1–inhibited rate/uninhibited rate)×100. Inhibitory activity of Haemangin and HlSGE on BSA proteolysis by trypsin and chymotrypsin was examined by pre-incubating the enzymes with Haemangin or HlSGE in a total reaction mixture of 40 µl for 1 h at 37°C for inhibition. Then, BSA (500 µg/ml) was added to inhibited and control (uninhibited) enzymes (250 µg/ml) and was further incubated overnight at 37°C. The tryptic digests were then analyzed by SDS-PAGE.

### Chick Aortic Ring Assay

A chick aortic ring assay was performed [15]. A twelve-day-old chick embryo was removed by cracking the egg; the thoracic cavity was opened and the heart and aortic arch were carefully removed. The aortic arch was cut into ∼1.0-mm pieces. A 96-well tissue culture plate was coated with 3 µl Matrigel/well (BD Biosciences) and aortic rings were placed into the wells; they were then held in place by overlaying 20 µl Matrigel/well. Then, 100 µl of EBM-2 (BD Biosciences) supplemented with antibiotics was added followed by the addition of Haemangin or HlSGE/OmSGE. A SG-specific recombinant protein from *H. longicornis*, peroxiredoxin (rTpx) [43], served as a negative control. Formation of vascular sprouts was observed for 3 days under a Leica microscope. A blind observer scored the density of vessel sprouts by comparing responses with media alone (control) to that observed with Haemangin. Results were scored as (i) 100%, sprout formation achieved with media alone (control); (ii) 75%, high levels of sprout formation; (iii) 50%, moderate levels of sprout formation; (iv) 25%, low levels of sprout formation; and (v) 10%, very low levels of sprout formation.

### CAM Assay

A chick chorioalantoic membrane (CAM) assay as an *in vivo* model of angiogenesis was performed to determine the antiangiogenic activity of Haemangin [44]. Haemangin or HlSGE in a total volume of 20 µl was loaded onto Thermanox discs, 13 mm in diameter (Nunc), and was applied to the CAM of 8-day-old embryos. PBS was used as a control. After 48 h of incubation at 37°C, a negative or positive response was assessed by two observers in a double-blind manner.

### Cell Culture and Capillary Tube Formation Assay

HUVECs (Cell Applications, Inc.) were cultured in growth medium at 37°C, 5% CO_2_ according to the manufacturer's instructions. Cells were incubated in T-25 tissue culture flasks to >80% confluency. Trypsinization was performed using the Subculturing Reagent Kit according to the manufacturer's instructions. Tube formation assay was performed in a 96-well plate coated with 50 µl Matrigel [45]. After trypsinization, HUVECs were seeded at 7.5×10^3^/well. Then, 100 µl/well growth medium was added. Cells were grown in the absence or presence of Haemangin or HlSGE/OmSGE at 37°C, 5% CO_2_. Tube formation was observed for 6 h under a Leica microscope. The number of tubes formed in individual wells was counted and data were expressed as a percentage compared to control.

### Cell Proliferation Assay

A cell proliferation assay was conducted using a cell proliferation assay kit (Promega) [46]. The 96-well plate was seeded with HUVECs at 3×10^3^/well. Cells were grown in 150 µl growth medium for 24 h at 37°C, 5% CO_2_. Then, medium was refreshed and Haemangin or HlSGE was added. After 3 days incubation, 30 µl MTS solution was added to each well. The plate was further incubated and the absorbance at 490 nm was measured using a spectrophotometer (Spectra Fluor). Results were expressed as percent inhibition of HUVEC proliferation in the presence of Haemangin or HlSGE compared to 100% cell proliferation achieved with growth medium alone. A cell cytotoxicity assay was performed using Cytotoxicity Detection Kit^PLUS^ (LDH) (Roche) as described by the manufacturer to determine the cytotoxic effect of Haemangin or HlSGE, if any.

### Apoptosis and Wound Healing Assay

Apoptosis was analyzed by Hoechst 33258 (Invitrogen) staining and by DNA-ladder assay. Hoechst 33258 staining of cells was performed by culturing HUVECs (1×10^5^ cells per well) onto glass coverslips in a 6-well plate (Sumitomo Bakelite) in growth medium at 37°C, 5% CO_2_. Cells were grown to 90% confluency. Then, Haemangin or HlSGE was added to the wells and cells were further incubated for 48 h at 37°C, 5% CO_2_. Cells were washed in PBS, fixed in 4% paraformaldehyde for 15 min, and stained with 8 µg/ml Hoechst 33258 (Hoechst) in H_2_O for 10 min. Cells were analyzed for nuclear morphologic changes using a Leica microscope equipped with fluorescence, DAPI, and composite filters (360/420 nm excitation and emission, respectively), and images were taken using Leica FW4000 Software. The DNA-ladder assay was carried out using the Apoptotic DNA Ladder Kit (Roche). Cells were cultured in a T-25 tissue culture flask (2.5×10^5^ cells/flask) in the absence or presence of Haemangin or HlSGE for 48 h at 37°C, 5% CO_2_ as mentioned above. The total cytoplasmic DNA was extracted from 2×10^6^ cells. Isolated DNA was analyzed using 1% agarose gel electrophoresis. The wound healing assay was performed in a 6-well plate [47]. HUVECs were seeded (1.25×10^5^ cells/well) in 6-well plate and were grown at 37°C, 5% CO_2_ to confluency. A wound was made by scratching a line across the monolayer of cells with a sterile pipette tip. The width of the cell-free gap was measured continuously under a Leica microscope by using Leica Application Suite. The response of wound healing was measured by the percentage reduction of the width of the scratch.

### Plasmin Inhibition Assay

Haemangin-stimulated plasmin degradation assay was performed [48]. Fibrinogen (8 µM; Sigma) and thrombin (0.83 NIH unit/ml; Sigma) were incubated in a 400 µl buffer containing 50 mM Tris-HCl (pH 7.5), 100 mM NaCl, and 5 mM CaCl_2_ at 25°C for 30 min. After the fibrin was completely polymerized, lysis of fibrin polymer was then initiated by adding 100 µl of buffer containing 20 µM plasminogen (Calbiochem) and 15 nM t-PA (Calbiochem) in the absence or presence of Haemangin or HlSGE. The reaction mixtures were then incubated overnight at 25°C and were subjected to SDS-PAGE. Plasmin degradation was examined by Coomassie staining of the proteins. To further examine the effect of Haemangin on fibrinolytic activity of plasmin, plasmin-dependent fibrinolysis inhibition assays were performed at 25°C to monitor changes in turbidity at 450 nm [48]. Fibrin polymer was prepared in a volume of 400 µl buffer and 1.5 µM plasmin (Sigma) without or with Haemangin, or HlSGE was added to initiate fibrinolysis. After the sample was incubated at 25°C for 12 h, plasmin-dependent fibrinolysis was observed and turbidity as function of plasmin was measured at an absorbance of 450 nm.

### RNA Interference

RNAi studies were carried out using dsRNA [42]. The coding sequence of Haemangin was cloned into pBluescript II SK+ plasmid (Toyobo). The dsRNA complementary to the *E. coli malE* gene was used as a negative control [49]. cDNA corresponding to *malE* mRNA was synthesized and was cloned into pBluescript II SK+ plasmid using the oligonucleotides 5′-CCGCTCGAGCGGTTATGAAAATAAAAACAGGTGCA-3′ and 5′-GAATTCGCTTGTCCTGGAACGCTTTGTC-3′ as forward and reverse primers, respectively. The inserted sequences of Haemangin and *malE* were amplified by PCR using the oligonucleotide T7 (5′-GTAATACGACTCACTATAGGGC-3′) and CM0422 primers (5′-GCGTAATACGACTCACTATAGGGAACAAAAGCTGGAGCT-3′) to attach T7 promoter recognition sites at both ends. The PCR products were purified using a gel extraction kit (Qiagen). dsRNA complementary to the DNA insert was synthesized by *in vitro* transcription using T7 RNA polymerase (Promega). One microgram each of Haemangin and *malE* dsRNA in 0.5 µl of PBS separately was injected into each unfed adult tick. Ticks were allowed to rest for 24 h at 25°C prior to placement on the host [16]. Rabbit tissues were also processed for H&E and silver nitrate staining.

### RNA Extraction and RT-PCR Analysis

The total RNA from tick SGs was isolated using an RNAeasy Mini Kit (Qiagen) and was submitted to reverse transcription (RT) before PCR. cDNA was synthesized and was employed to perform PCRs using either Haemangin-specific oligonucleotides (5′-CATTTCGCCAACCATCTTTC-3′ and 5′-TGACAGGTCCAGCAGCTATG-3′) or oligonucleotides specific for β-actin. Quantitative RT-PCR was done using LightCycler FastStart DNA Master SYBR Green I (Roche) in a LightCycler 1.5 instrument (Roche) [16].

### RNA Extraction and Microarray Analysis

To perform oligonucleotide microarray analysis, HUVECs were cultured in the absence (control) or presence of Haemangin (100 nM) for 48 h as described above. Approximately 2×10^6^ cells were harvested and the total RNA was extracted as described above. The mRNAs were then prepared and analyzed by hybridization to microarrays (Filgen Array Human35k, oligo DNA microarray).

### Statistical Analysis

Data are reported as means±standard errors, where appropriate. The statistical significance (*p*<0.05) was determined by Student's *t* test/Mann-Whitney's U test.

### Nucleotide Sequence Accession Number

The nucleotide sequence data reported in this paper will appear in the DDBJ/EMBL/GENBANK nucleotide sequence databases with the accession number AB434485.

## Supporting Information

Figure S1 Cluster of up-regulated genes. Microarray analysis was performed using total RNA extracted from HUVECs treated with Haemangin for 48 h. The total numbers of genes filtered to include only those with ≧2.0 fold up-regulated compared with untreated control are categorized according to their molecular functions and are indicated with their relative numbers.
(0.05 MB PDF)

Figure S2 Cluster of down-regulated genes. Total RNA was extracted from HUVECs treated with Haemangin for 48 h and was subjected to microarray analysis. The ≧2.0 fold down-regulated genes compared with untreated control are categorized according to their molecular functions and are indicated with their relative numbers.
(0.03 MB PDF)

Table S1 Haemangin-induced up-regulated genes ordered into different biological processes. Transcripts were filtered to include only those with ≧2.0 fold changes compared with untreated control.
(0.10 MB PDF)

Table S2 Haemangin-induced down-regulated genes ordered into different biological processes. Transcripts were filtered to include only those with ≧2.0 fold changes compared with untreated control.
(0.08 MB PDF)
