# Supplementary figures and images for: The Kunitz-Like Modulatory Protein Haemangin Is Vital for Hard Tick Blood-Feeding Success
Source: PLoS Pathog. 2009 Jul 10;5(7):e1000497. doi: 10.1371/journal.ppat.1000497 (PMC2701603; doi:10.1371/journal.ppat.1000497)

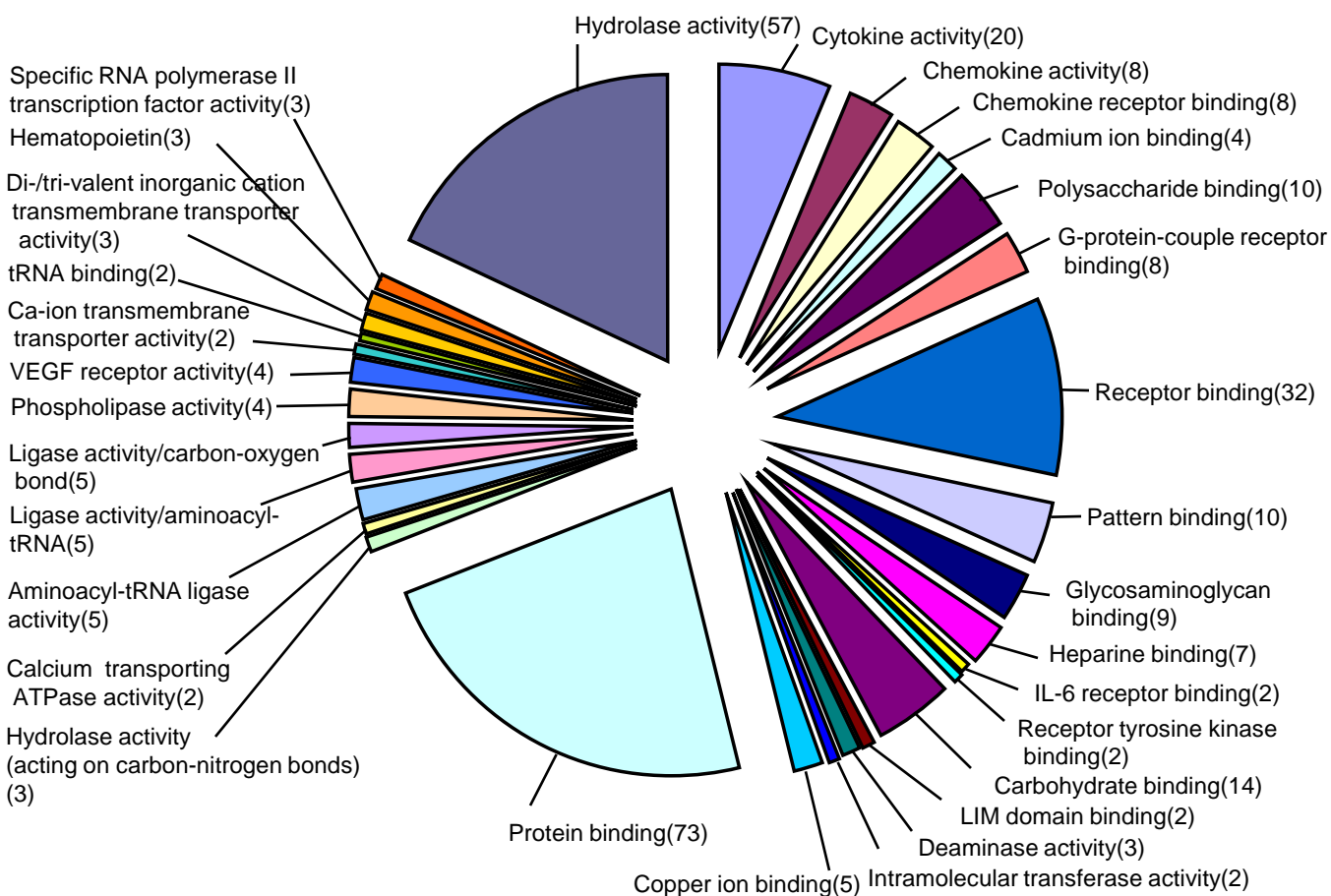

Supplement: Figure S1 — Cluster of up-regulated genes. Microarray analysis was performed using total RNA extracted from HUVECs treated with Haemangin for 48 h. The total numbers of genes filtered to include only those with ≧2.0 fold up-regulated compared with untreated control are categorized according to their molecular functions and are indicated with their relative numbers. (0.05 MB PDF) [file ppat.1000497.s001.pdf]

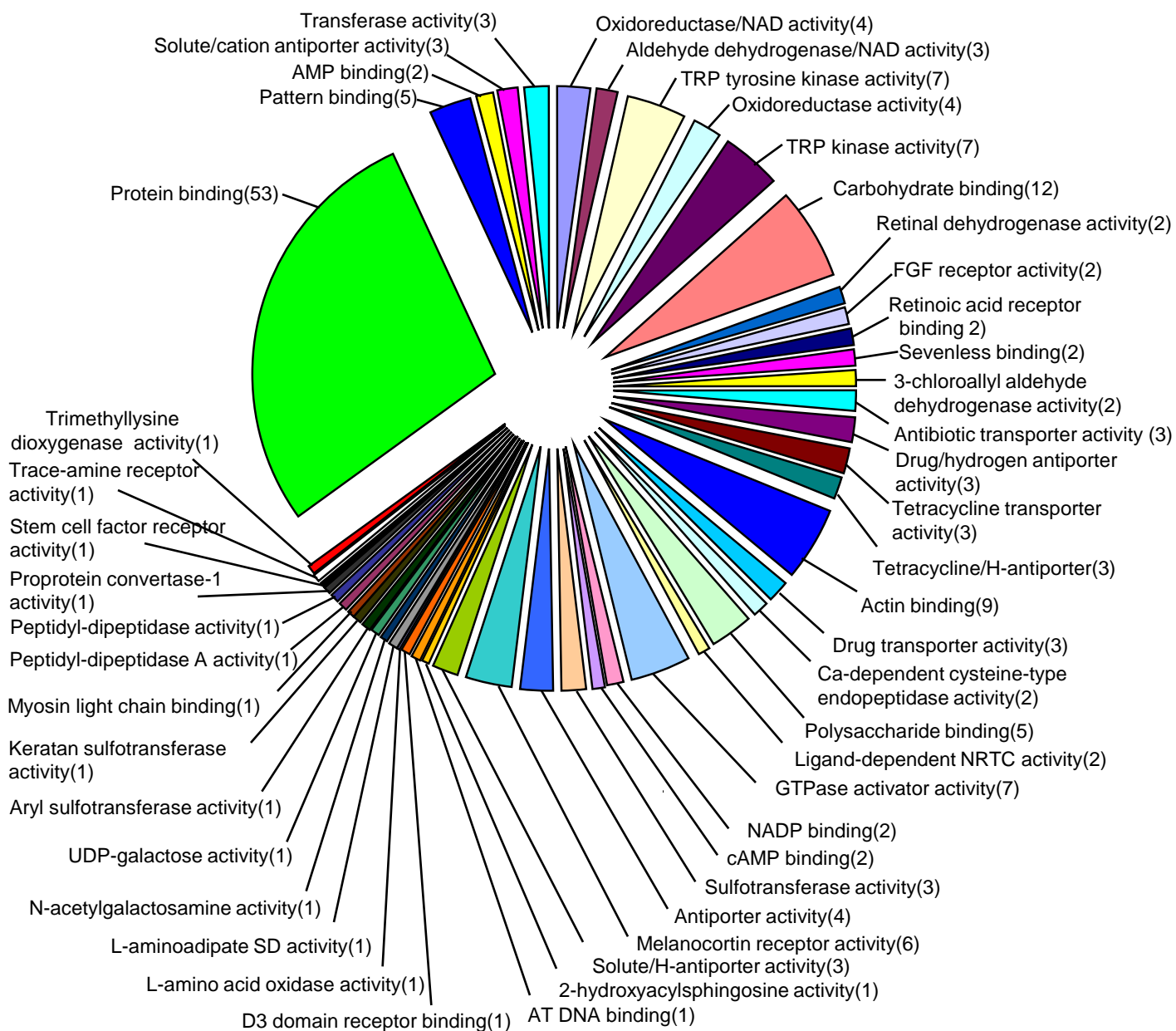

Supplement: Figure S2 — Cluster of down-regulated genes. Total RNA was extracted from HUVECs treated with Haemangin for 48 h and was subjected to microarray analysis. The ≧2.0 fold down-regulated genes compared with untreated control are categorized according to their molecular functions and are indicated with their relative numbers. (0.03 MB PDF) [file ppat.1000497.s002.pdf]
