# Supplementary material for: The Kunitz-Like Modulatory Protein Haemangin Is Vital for Hard Tick Blood-Feeding Success
Source: PLoS Pathog. 2009 Jul 10;5(7):e1000497. doi: 10.1371/journal.ppat.1000497 (PMC2701603; doi:10.1371/journal.ppat.1000497)
Supplement: Table S1 — Haemangin-induced up-regulated genes ordered into different biological processes. Transcripts were filtered to include only those with ≧2.0 fold changes compared with untreated control. (0.10 MB PDF) [file ppat.1000497.s003.pdf]

Term	Changed Genes	Total Genes	Z score	P-value
immune system process	51(0)	613(0)	8.532	2.45E-12
immune response	40(30)	455(313)	7.971	1.79E-10
response to stimulus	83(0)	1682(0)	5.589	4.09E-08
defense response	26(0)	288(0)	6.59	2.23E-07
response to wounding	30(0)	384(0)	6.144	4.63E-07
inflammatory response	24(18)	261(175)	6.441	4.92E-07
response to external stimulus	37(0)	582(6)	5.403	2.98E-06
regulation of apoptosis	27(5)	417(81)	4.722	0.0000497
negative regulation of developmental process	21(0)	282(0)	4.888	0.0000516
regulation of programmed cell death	27(0)	423(0)	4.64	0.0000605
regulation of developmental process	35(1)	626(2)	4.425	0.0000739
negative regulation of apoptosis	16(4)	185(49)	4.965	0.0000828
myeloid cell differentiation	10(0)	76(10)	5.602	0.0000846
negative regulation of programmed cell death	16(0)	188(2)	4.889	0.0000987
response to chemical stimulus	28(0)	472(4)	4.299	0.000151
apoptosis	33(14)	597(276)	4.226	0.000152
response to biotic stimulus	12(0)	117(3)	5.019	0.000152
programmed cell death	33(0)	603(1)	4.164	0.000171
cell death	34(1)	632(30)	4.119	0.000222
death	34(0)	632(0)	4.119	0.000222
response to other organism	9(0)	71(0)	5.165	0.000247
anti-apoptosis	12(9)	130(106)	4.571	0.000372
chemotaxis	13(12)	152(131)	4.428	0.000424
taxis	13(0)	152(0)	4.428	0.000424
cell activation during immune response	4(0)	11(0)	6.867	0.000517
leukocyte activation during immune response	4(0)	11(0)	6.867	0.000517
morphogenesis of an epithelial sheet	3(3)	4(4)	8.896	0.00059
leukocyte activation	12(0)	142(2)	4.205	0.000774
regulation of cell proliferation	23(2)	393(33)	3.826	0.000794
response to stress	44(3)	964(79)	3.529	0.000819
hemopoiesis	12(2)	145(27)	4.12	0.000918
immune system development	13(0)	171(0)	3.932	0.00118
cytokine production	9(1)	92(11)	4.171	0.00137
locomotory behavior	14(0)	199(29)	3.75	0.00155
hemopoietic or lymphoid organ development	12(0)	155(0)	3.851	0.00157
cell development	42(0)	943(11)	3.282	0.0016
positive regulation of nitric oxide biosynthetic process	3(3)	7(7)	6.536	0.00191
positive regulation of nitrogen compound metabolic process	3(0)	7(0)	6.536	0.00191
behavior	18(1)	300(17)	3.5	0.0021
cell activation	12(0)	162(2)	3.675	0.00222
regulation of cell activation	8(0)	82(0)	3.923	0.00255
regulation of leukocyte activation	8(0)	82(0)	3.923	0.00255
I-kappaB kinase/NF-kappaB cascade	9(0)	102(17)	3.796	0.00265
cell proliferation	29(6)	595(220)	3.239	0.00275
cell differentiation	55(9)	1357(266)	3.028	0.00294
cellular developmental process	55(0)	1357(0)	3.028	0.00294
regulation of defense response	5(0)	33(0)	4.396	0.00305
negative regulation of mast cell cytokine production	2(2)	2(2)	8.466	0.00403
negative regulation of viral genome replication	2(1)	2(1)	8.466	0.00403
negative regulation of viral reproduction	2(1)	2(1)	8.466	0.00403
positive regulation of lymphocyte activation	6(0)	52(0)	3.915	0.00412
CD4-positive/, alpha-beta T cell differentiation during immune response	3(0)	10(0)	5.31	0.00429
T cell activation during immune response	3(0)	10(0)	5.31	0.00429
T cell differentiation during immune response	3(0)	10(0)	5.31	0.00429
T-helper cell differentiation	3(0)	10(1)	5.31	0.00429
alpha-beta T cell differentiation during immune response	3(0)	10(0)	5.31	0.00429
lymphocyte activation during immune response	3(0)	10(0)	5.31	0.00429
regulation of nitric oxide biosynthetic process	3(0)	10(1)	5.31	0.00429
response to virus	6(5)	53(44)	3.855	0.00449
alpha-beta T cell activation	4(0)	22(0)	4.464	0.00451
regulation of I-kappaB kinase/NF-kappaB cascade	7(1)	73(1)	3.614	0.00511
positive regulation of cell activation	6(0)	56(0)	3.684	0.00573
positive regulation of leukocyte activation	6(0)	56(0)	3.684	0.00573
regulation of lymphocyte activation	7(0)	75(0)	3.527	0.00585

myeloid leukocyte activation	4(0)	24(0)	4.206	0.00592
CD4-positive/, alpha beta T cell differentiation	3(0)	12(0)	4.75	0.00656
L-serine biosynthetic process	2(2)	3(3)	6.816	0.0066
arachidonic acid metabolic process	2(1)	3(1)	6.816	0.0066
establishment of epithelial cell polarity	2(2)	3(3)	6.816	0.0066
myeloid dendritic cell differentiation	2(2)	3(3)	6.816	0.0066
negative regulation of cytokine production during immune response	2(0)	3(0)	6.816	0.0066
negative regulation of production of molecular mediator of immune response	2(0)	3(0)	6.816	0.0066
polarized epithelial cell differentiation	2(0)	3(0)	6.816	0.0066
calcium-independent cell-cell adhesion	3(2)	13(12)	4.518	0.00792
lymphocyte activation	9(0)	123(3)	3.141	0.00824
developmental process	82(0)	2324(0)	2.406	0.00846
negative regulation of cell proliferation	11(10)	170(137)	3.013	0.00856
positive regulation of developmental process	16(0)	285(0)	3.011	0.00882
mast cell cytokine production	2(0)	4(0)	5.82	0.00973
regulation of T-helper 2 cell differentiation	2(0)	4(0)	5.82	0.00973
regulation of mast cell cytokine production	2(0)	4(0)	5.82	0.00973
regulation of cell adhesion	5(2)	45(16)	3.466	0.00997
nitrogen compound metabolic process	18(0)	342(14)	2.899	0.01046
cytokine metabolic process	6(1)	65(1)	3.233	0.01098
carboxylic acid metabolic process	20(0)	398(11)	2.835	0.01129
organic acid metabolic process	20(0)	401(0)	2.799	0.01164
homeostasis of number of cells	5(0)	47(0)	3.343	0.01172
lymphocyte proliferation	5(1)	47(3)	3.343	0.01172
mononuclear cell proliferation	5(0)	47(0)	3.343	0.01172
erythrocyte differentiation	4(0)	30(13)	3.579	0.01186
erythrocyte homeostasis	4(0)	30(0)	3.579	0.01186
regulation of immune system process	9(0)	131(0)	2.926	0.01188
regulation of multicellular organismal process	15(0)	267(0)	2.918	0.01206
regulation of alpha-beta T cell activation	3(0)	16(0)	3.947	0.01292
regulation of nitrogen compound metabolic process	3(0)	16(0)	3.947	0.01292
myeloid leukocyte differentiation	4(0)	31(0)	3.491	0.01311
positive regulation of apoptosis	12(4)	198(34)	2.897	0.01324
T-helper 2 cell differentiation	2(0)	5(1)	5.13	0.01338
establishment of apical/basal cell polarity	2(0)	5(2)	5.13	0.01338
myeloid dendritic cell activation	2(0)	5(0)	5.13	0.01338
negative regulation of bone mineralization	2(2)	5(5)	5.13	0.01338
negative regulation of cytokine production	2(0)	5(0)	5.13	0.01338
positive regulation of programmed cell death	12(0)	200(1)	2.858	0.01378
amino acid metabolic process	12(1)	203(38)	2.802	0.01469
nitric oxide biosynthetic process	3(0)	17(8)	3.789	0.01491
nitric oxide metabolic process	3(0)	17(0)	3.789	0.01491
regulation of inflammatory response	3(2)	17(4)	3.789	0.01491
di-/, tri-valent inorganic cation transport	8(0)	114(1)	2.827	0.01546
positive regulation of cellular process	35(0)	857(0)	2.464	0.01587
regulation of myeloid cell differentiation	4(0)	33(1)	3.325	0.01588
leukocyte differentiation	6(0)	72(0)	2.934	0.01692
alpha-beta T cell differentiation	3(0)	18(1)	3.642	0.01707
L-serine metabolic process	2(0)	6(3)	4.615	0.01754
negative regulation of immune effector process	2(0)	6(0)	4.615	0.01754
phospholipid catabolic process	2(2)	6(4)	4.615	0.01754
regulation of Notch signaling pathway	2(0)	6(2)	4.615	0.01754
response to lipopolysaccharide	2(2)	6(6)	4.615	0.01754
multi-organism process	11(0)	179(0)	2.824	0.01792
cellular cation homeostasis	9(0)	142(0)	2.657	0.01867
negative regulation of multicellular organismal process	6(0)	74(0)	2.855	0.01895
nitrogen compound biosynthetic process	6(0)	74(0)	2.855	0.01895
positive regulation of cell proliferation	12(7)	214(158)	2.603	0.01902
cell motility	17(3)	346(105)	2.516	0.01913
localization of cell	17(0)	346(0)	2.516	0.01913
immune effector process	8(0)	119(0)	2.69	0.01925
DNA alkylation	3(0)	19(0)	3.507	0.01938
DNA methylation	3(2)	19(16)	3.507	0.01938
maintenance of protein localization	3(0)	19(0)	3.507	0.01938
positive regulation of B cell activation	3(0)	20(0)	3.381	0.02186
regulation of immune effector process	3(0)	20(0)	3.381	0.02186
adaptive immune response	7(0)	99(1)	2.667	0.02207
adaptive immune response based on somatic recombination of immune recepto	7(0)	99(0)	2.667	0.02207

erythrocyte development	2(2)	7(2)	4.21	0.02216
establishment of cell polarity	2(0)	7(0)	4.21	0.02216
regulation of T-helper cell differentiation	2(0)	7(0)	4.21	0.02216
response to exogenous dsRNA	2(2)	7(7)	4.21	0.02216
serine family amino acid biosynthetic process	2(0)	7(0)	4.21	0.02216
regulation of cell motility	5(0)	56(2)	2.861	0.02218
system development	49(0)	1319(0)	2.232	0.02305
viral genome replication	3(1)	21(11)	3.263	0.02451
cellular di-/, tri-valent inorganic cation homeostasis	8(0)	125(0)	2.535	0.02463
di-/, tri-valent inorganic cation homeostasis	8(0)	125(0)	2.535	0.02463
metal ion transport	16(1)	327(16)	2.423	0.02469
cation transport	18(0)	382(0)	2.401	0.02486
regulation of cytokine biosynthetic process	5(0)	58(4)	2.768	0.02513
positive regulation of biological process	36(0)	916(0)	2.261	0.02538
blood vessel development	10(2)	162(26)	2.708	0.02545
vasculature development	10(0)	163(0)	2.687	0.02579
response to hypoxia	4(4)	39(39)	2.898	0.02626
chemokine biosynthetic process	2(0)	8(0)	3.879	0.02722
chemokine metabolic process	2(0)	8(0)	3.879	0.02722
chemokine production	2(0)	8(0)	3.879	0.02722
cytokine production during immune response	2(0)	8(0)	3.879	0.02722
cytoplasmic sequestering of transcription factor	2(1)	8(5)	3.879	0.02722
interleukin-12 biosynthetic process	2(0)	8(0)	3.879	0.02722
lipopolysaccharide-mediated signaling pathway	2(2)	8(8)	3.879	0.02722
morphogenesis of a polarized epithelium	2(0)	8(1)	3.879	0.02722
regulation of cell-cell adhesion	2(0)	8(0)	3.879	0.02722
regulation of chemokine biosynthetic process	2(0)	8(0)	3.879	0.02722
regulation of cytokine production during immune response	2(0)	8(0)	3.879	0.02722
regulation of interleukin-12 biosynthetic process	2(0)	8(1)	3.879	0.02722
regulation of viral genome replication	2(0)	8(3)	3.879	0.02722
response to dsRNA	2(0)	8(0)	3.879	0.02722
DNA modification	3(0)	22(3)	3.152	0.02732
cellular metal ion homeostasis	7(0)	104(5)	2.52	0.02757
metal ion homeostasis	7(0)	104(0)	2.52	0.02757
regulation of lymphocyte proliferation	4(0)	40(0)	2.835	0.0283
regulation of mononuclear cell proliferation	4(0)	40(0)	2.835	0.0283
translation	10(0)	172(0)	2.501	0.02989
amino acid activation	5(0)	61(0)	2.635	0.02999
regulation of locomotion	5(0)	61(3)	2.635	0.02999
tRNA aminoacylation	5(0)	61(0)	2.635	0.02999
tRNA aminoacylation for protein translation	5(4)	61(50)	2.635	0.02999
amino acid and derivative metabolic process	13(0)	253(7)	2.372	0.03044
cellular chemical homeostasis	10(0)	174(0)	2.461	0.03106
cellular ion homeostasis	10(0)	174(4)	2.461	0.03106
calcium ion transport	7(7)	108(98)	2.409	0.03258
T-helper 2 type immune response	2(1)	9(3)	3.601	0.0327
cytoplasmic sequestering of protein	2(0)	9(0)	3.601	0.0327
defense response to virus	2(1)	9(5)	3.601	0.0327
negative regulation of ossification	2(0)	9(4)	3.601	0.0327
positive regulation of B cell proliferation	2(2)	9(9)	3.601	0.0327
regulation of CD4-positive/, alpha beta T cell differentiation	2(0)	9(0)	3.601	0.0327
regulation of production of molecular mediator of immune response	2(0)	9(0)	3.601	0.0327
regulation of cytokine production	3(0)	24(3)	2.95	0.03344
response to antibiotic	3(3)	24(24)	2.95	0.03344
angiogenesis	7(6)	109(70)	2.382	0.03393
amine metabolic process	15(0)	314(5)	2.248	0.03493
cytokine biosynthetic process	5(0)	64(0)	2.509	0.03542
locomotion	5(0)	64(2)	2.509	0.03542
positive regulation of cellular biosynthetic process	5(0)	64(0)	2.509	0.03542
biological adhesion	25(0)	591(0)	2.265	0.03639
cell adhesion	25(17)	591(423)	2.265	0.03639
cellular homeostasis	12(0)	227(5)	2.384	0.03657
positive regulation of biosynthetic process	5(0)	65(0)	2.469	0.03735
G1 phase	2(1)	10(1)	3.364	0.03856
Notch signaling pathway	2(0)	10(0)	3.364	0.03856
establishment and/or maintenance of epithelial cell polarity	2(0)	10(6)	3.364	0.03856
interleukin-12 production	2(0)	10(1)	3.364	0.03856
regulation of erythrocyte differentiation	2(0)	10(0)	3.364	0.03856

organ development	37(0)	977(0)	2.06	0.03873
negative regulation of cell differentiation	5(2)	66(14)	2.43	0.03935
regulation of DNA binding	4(0)	45(3)	2.549	0.03992
negative regulation of biological process	35(0)	909(0)	2.105	0.04168
negative regulation of cell activation	3(0)	27(0)	2.685	0.04382
negative regulation of leukocyte activation	3(0)	27(0)	2.685	0.04382
T-helper 1 type immune response	2(1)	11(6)	3.157	0.0448
negative regulation of protein import into nucleus	2(0)	11(0)	3.157	0.0448
negative regulation of transcription factor import into nucleus	2(0)	11(1)	3.157	0.0448
regulation of isotype switching	2(0)	11(2)	3.157	0.0448
regulation of viral reproduction	2(0)	11(0)	3.157	0.0448
response to molecule of bacterial origin	2(0)	11(3)	3.157	0.0448
cation homeostasis	9(0)	160(0)	2.265	0.04567
positive regulation of I-kappaB kinase/NF-kappaB cascade	5(5)	69(69)	2.316	0.04573
maintenance of localization	3(0)	28(0)	2.605	0.0476
regulation of cell migration	4(3)	48(18)	2.395	0.04801
