# Supplementary material for: The Kunitz-Like Modulatory Protein Haemangin Is Vital for Hard Tick Blood-Feeding Success
Source: PLoS Pathog. 2009 Jul 10;5(7):e1000497. doi: 10.1371/journal.ppat.1000497 (PMC2701603; doi:10.1371/journal.ppat.1000497)
Supplement: Table S2 — Haemangin-induced down-regulated genes ordered into different biological processes. Transcripts were filtered to include only those with ≧2.0 fold changes compared with untreated control. (0.08 MB PDF) [file ppat.1000497.s004.pdf]

Term	Changed Genes	Total Genes	Z score	P-value
mitosis	11(9)	155(105)	4.435	0.000507
cell cycle process	15(0)	265(0)	4.149	0.000511
M phase of mitotic cell cycle	11(1)	159(8)	4.333	0.000619
M phase	12(0)	194(1)	4.066	0.000901
cytoskeleton organization and biogenesis	18(4)	377(55)	3.734	0.00126
cell cycle phase	13(0)	252(0)	3.484	0.00263
mitotic cell cycle	12(0)	229(6)	3.408	0.00338
aldehyde metabolic process	3(3)	14(10)	5.118	0.0044
anatomical structure morphogenesis	26(2)	714(83)	3.002	0.00574
cell cycle	20(13)	521(336)	2.882	0.00705
regulation of MAP kinase activity	6(0)	80(2)	3.441	0.00755
mitotic cell cycle spindle assembly checkpoint	2(2)	5(5)	5.99	0.00788
blood vessel morphogenesis	8(0)	140(4)	3.061	0.00987
spindle checkpoint	2(0)	6(1)	5.409	0.01037
tetracycline transport	3(3)	23(23)	3.722	0.01475
cell division	8(7)	151(137)	2.818	0.01476
cortical actin cytoskeleton organization and biogenesis	2(2)	8(8)	4.582	0.01623
response to antibiotic	3(3)	24(24)	3.614	0.01636
cell proliferation	21(8)	595(220)	2.548	0.01648
cell communication	80(1)	3103(73)	2.074	0.01691
morphogenesis of an epithelium	5(2)	71(8)	2.97	0.01815
cholesterol homeostasis	2(2)	9(9)	4.272	0.01958
cortical cytoskeleton organization and biogenesis	2(0)	9(1)	4.272	0.01958
lipid homeostasis	2(0)	9(0)	4.272	0.01958
sterol homeostasis	2(0)	9(0)	4.272	0.01958
regulation of cell migration	4(2)	48(18)	3.073	0.02016
MAPKKK cascade	7(2)	130(17)	2.684	0.02036
blood vessel development	8(2)	162(26)	2.596	0.02121
regulation of mitosis	4(0)	49(7)	3.021	0.02147
drug transport	3(0)	27(2)	3.324	0.02173
response to drug	3(0)	27(0)	3.324	0.02173
vasculature development	8(0)	163(0)	2.576	0.02188
pancreas development	2(1)	10(3)	4.007	0.02319
myoblast maturation	2(0)	11(0)	3.777	0.02705
signal transduction	73(44)	2849(1482)	1.928	0.02738
angiogenesis	6(4)	109(70)	2.546	0.02815
forebrain development	4(3)	55(28)	2.735	0.03044
positive regulation of MAP kinase activity	4(1)	55(6)	2.735	0.03044
myoblast development	2(0)	12(1)	3.574	0.03115
negative regulation of angiogenesis	2(2)	12(12)	3.574	0.03115
protein export from nucleus	2(1)	12(8)	3.574	0.03115
striated muscle cell differentiation	2(0)	12(3)	3.574	0.03115
regulation of cell motility	4(0)	56(2)	2.691	0.03212
regulation of protein kinase activity	8(0)	177(2)	2.32	0.03292
regulation of cell cycle	10(0)	238(0)	2.344	0.034
cell motility	13(3)	346(105)	2.242	0.03401
localization of cell	13(0)	346(0)	2.242	0.03401
organ morphogenesis	13(2)	346(101)	2.242	0.03401
actomyosin structure organization and biogenesis	2(1)	13(8)	3.394	0.03547
monocarboxylic acid transport	2(1)	13(5)	3.394	0.03547
transmembrane receptor protein tyrosine kinase signaling pathway	7(4)	149(62)	2.282	0.03767
ADP biosynthetic process	1(1)	1(1)	6.914	0.03975
DNA ligation during DNA repair	1(0)	1(0)	6.914	0.03975
MAPK export from nucleus	1(1)	1(1)	6.914	0.03975
MAPK phosphatase export from nucleus	1(0)	1(0)	6.914	0.03975
MAPK phosphatase export from nucleus/, leptomycin B sensitive	1(1)	1(1)	6.914	0.03975
base-excision repair/, DNA ligation	1(1)	1(1)	6.914	0.03975
cell wall chitin metabolic process	1(1)	1(1)	6.914	0.03975
cell wall polysaccharide metabolic process	1(0)	1(0)	6.914	0.03975
centrosome separation	1(1)	1(1)	6.914	0.03975
cerebellar Purkinje cell layer development	1(1)	1(1)	6.914	0.03975
cocaine metabolic process	1(1)	1(1)	6.914	0.03975
cytokinesis after mitosis	1(1)	1(1)	6.914	0.03975
cytokinesis during cell cycle	1(0)	1(0)	6.914	0.03975
dADP biosynthetic process	1(1)	1(1)	6.914	0.03975

dADP metabolic process	1(0)	1(0)	6.914	0.03975
deoxyribonucleoside diphosphate biosynthetic process	1(0)	1(0)	6.914	0.03975
fourth ventricle development	1(1)	1(1)	6.914	0.03975
loss of chromatin silencing	1(1)	1(1)	6.914	0.03975
negative regulation of vasodilation	1(1)	1(1)	6.914	0.03975
nucleoside diphosphate biosynthetic process	1(0)	1(0)	6.914	0.03975
positive regulation of striated muscle cell differentiation	1(1)	1(1)	6.914	0.03975
progesterone biosynthetic process	1(1)	1(1)	6.914	0.03975
purine deoxyribonucleoside diphosphate biosynthetic process	1(0)	1(0)	6.914	0.03975
purine deoxyribonucleoside diphosphate metabolic process	1(0)	1(0)	6.914	0.03975
purine deoxyribonucleotide biosynthetic process	1(0)	1(0)	6.914	0.03975
purine nucleoside diphosphate biosynthetic process	1(0)	1(0)	6.914	0.03975
purine ribonucleoside diphosphate biosynthetic process	1(0)	1(0)	6.914	0.03975
regulation of striated muscle cell differentiation	1(0)	1(0)	6.914	0.03975
release of virus from host	1(1)	1(1)	6.914	0.03975
ribonucleoside diphosphate biosynthetic process	1(0)	1(0)	6.914	0.03975
third ventricle development	1(1)	1(1)	6.914	0.03975
ventricular cardiac muscle cell development	1(1)	1(1)	6.914	0.03975
ventricular cardiac muscle cell differentiation	1(0)	1(0)	6.914	0.03975
viral assembly/, maturation/, egress/, and release	1(0)	1(0)	6.914	0.03975
regulation of locomotion	4(0)	61(3)	2.485	0.04131
DNA duplex unwinding	2(0)	15(0)	3.085	0.04476
DNA geometric change	2(0)	15(0)	3.085	0.04476
DNA unwinding during replication	2(2)	15(15)	3.085	0.04476
fibroblast growth factor receptor signaling pathway	2(2)	15(12)	3.085	0.04476
negative regulation of cell migration	2(2)	15(14)	3.085	0.04476
actin cytoskeleton organization and biogenesis	7(2)	157(71)	2.131	0.04721
locomotion	4(0)	64(2)	2.372	0.04748
negative regulation of catalytic activity	4(0)	65(3)	2.336	0.04964
inactivation of MAPK activity	2(2)	16(15)	2.951	0.0497
